# Assessment of Inequalities in Coverage of Essential Reproductive, Maternal, Newborn, Child, and Adolescent Health Interventions in Kenya

**DOI:** 10.1001/jamanetworkopen.2018.5152

**Published:** 2018-12-28

**Authors:** Emily Catherine Keats, Nadia Akseer, Zaid Bhatti, William Macharia, Anthony Ngugi, Arjumand Rizvi, Zulfiqar Ahmed Bhutta

**Affiliations:** 1 Centre for Global Child Health, The Hospital for Sick Children, Toronto, Ontario, Canada; 2 Dalla Lana School of Public Health, University of Toronto, Toronto, Ontario, Canada; 3 Aga Khan University, Karachi, Pakistan; 4 Aga Khan University, Nairobi, Kenya

## Abstract

**Importance:**

Previous work has underscored subnational inequalities that could impede additional health gains in Kenya.

**Objective:**

To provide a comprehensive assessment of the burden, distribution, and change in inequalities in reproductive, maternal, newborn, child, and adolescent health (RMNCAH) interventions in Kenya from 2003 to 2014.

**Design, Setting, and Participants:**

This population-based cross-sectional study used data from the 2003, 2008, and 2014 Kenya Demographic and Health Surveys. The study included women of reproductive age (ages 15-49 years) and children younger than years, with national, regional, county, and subcounty level representation. Data analysis was conducted from April 2018 to November 2018.

**Exposures:**

Socioeconomic position that was derived from asset indices and presented as wealth quintiles. Urban and rural residence and regions of Kenya were also considered.

**Main Outcomes and Measures:**

Absolute and relative measures of inequality in coverage of RMNCAH interventions.

**Results:**

For this analysis, representative samples of 31 380 women of reproductive age and 29 743 children younger than 5 years from across Kenya were included. The RMNCAH interventions examined demonstrated pro-rich and bottom inequality patterns. The most inequitable interventions were skilled birth attendance, family planning needs satisfied, and 4 or more antenatal care visits, whereby the absolute difference in coverage between the wealthiest (quintile 5) and poorest quintiles (quintile 1) was 61.6% (95% CI, 60.1%-63.1%), 33.4% (95% CI, 31.9%-34.9%), and 31.0% (95% CI, 30.5%-31.6%), respectively. The most equitable intervention was early initiation of breastfeeding, with an absolute difference (quintile 5 minus quintile 1) of −7.9% (95% CI, −11.1% to −4.8%), although antenatal care (1 visit) and diphtheria-tetanus-pertussis immunization (3 doses) demonstrated the best combination of high coverage and low inequalities. Our geospatial analysis revealed significant socioeconomic disparities in the northern and eastern regions of Kenya that have translated to suboptimal intervention coverage. A significant gap remains for rural, disadvantaged populations.

**Conclusions and Relevance:**

Coverage of RMNCAH interventions has improved over time, but wealth and geospatial inequalities in Kenya are persistent. Policy and programming efforts should place more emphasis on improving the accessibility of health facility-based interventions, which generally demonstrate poor coverage and high inequalities, and focus on integrated approaches to maternal health service delivery at the community level when access is poor. Scaling up of health services for the urban and, in particular, rural poor areas and those residing in Kenya’s former north eastern province will contribute toward achievement of universal health coverage.

## Key Points

**Question:**

How equitable was coverage of essential reproductive, maternal, newborn, child, and adolescent health interventions in Kenya throughout the Millennium Development Goal period?

**Findings:**

In this population-based, cross-sectional study of 31 380 women of reproductive age and 29 743 children, regional and socioeconomic inequalities in coverage of several key interventions persisted throughout the study period (2003-2014), placing both the rural and urban poor as well as populations in the northern and eastern regions of Kenya at a significant disadvantage.

**Meaning:**

For Kenya to improve health equity and achieve its 2030 goals, a targeted approach will be needed to reach populations that are currently lagging behind.

## Teaser

This population-based cross-sectional study of data from the Kenya Demorgraphic and Health Surveys (2003, 2008, and 2014) assesses the burden, distribution, and change in inequalities in reproductive, maternal, newborn, child, and adolescent health interventions in Kenya.

## Introduction

The Millennium Development Goal era saw notable improvements in maternal and child health globally.^1^ Kenya—a rapidly developing hub of the east African economic block—benefitted substantially from a focused agenda on reproductive, maternal, newborn, child and adolescent health (RMNCAH).^2^

Although the 1990s were plagued by a sharp increase in mortality, which was attributed largely to the HIV epidemic and financial and social conditions, the country managed to rapidly reverse the trend through targeted and widespread policies and programs for RMNCAH. Impressively, the maternal mortality ratio dropped 39%, from 590 deaths per 100 000 live births in 1998 to 362 per 100 000 live births in 2014.^2^ Over the same period, the under-five mortality rate declined by 54% (from 112 to 52 deaths/1000 live births) and neonatal mortality rate by 21% (from 28 to 22 deaths/1000 live births).^2^ Gains at the national level, however, were compounded by huge subnational inequalities. Keats et al^2^ suggested that socioeconomic and regional inequalities pose a threat to further RMNCAH gains, and a targeted focus on promoting equity was critical for the success of the joint drive by government and development partners to attain universal health coverage.^3^ Establishing equity is also central to the success of the Sustainable Development Goal agenda, to which the government of Kenya has pledged through several national policy documents.^4,5^

To identify those populations that are truly lagging behind and to benchmark equality gains, a comprehensive overview of the burden, distribution, and trends in inequality in Kenya are essential. The concepts of equity and equality differ, and should be understood for the purpose of this study. Equity refers the degree of fairness or justice in a society, while inequalities (in coverage or service access) can be measured, and may increase pervasive societal inequities.^6,7^ To date, few studies have provided a systematic evaluation of inequalities in RMNCAH intervention coverage in Kenya. A handful of studies have demonstrated both socioeconomic and regional inequalities in relation to intervention coverage,^8,9^ access to health services,^10,11,12,13^ and provision of health services^14,15^ in the country. However, none have examined the complete set of essential interventions across the continuum of care, nor have they characterized the magnitude of inequality through both the absolute and relative lenses. Additionally, to our knowledge, no other studies have examined the range of equity dimensions as relevant to the Kenyan context, including wealth status, urban and rural disaggregation, and geographical disparities at the regional, county, and subcounty level.

This novel analysis aims to provide a comprehensive assessment of the burden, distribution, and change in inequalities in RMNCAH interventions in Kenya from 2003 to 2014. Using data from nationally representative surveys, we explored the coverage and relative and absolute socioeconomic inequalities of 11 essential RMNCAH interventions at both the national and subnational level.

## Methods

### Data Sources

We used data from the 2014 Kenya Demographic and Health Survey (K-DHS), the most recent, large-scale national survey conducted in Kenya that sampled 14 741 women of reproductive age (ages 15-49 years) and 18 702 children younger than 5 years.^16^ This survey is powered at the subnational (county) level and provides estimates for maternal and child health and nutrition indicators across the continuum of care. The K-DHS also contains comprehensive information on household assets that were used to compute wealth indices. For trend analyses, we included data from the 2003 and 2008 K-DHS surveys, which each sampled 5560 and 5481 children under the age of 5 years and 8195 and 8444 women of reproductive age, respectively. This study follows the Strengthening the Reporting of Observational Studies in Epidemiology (STROBE) reporting guideline. As this study consisted of secondary data analysis only, ethical review was waived. All ethics procedures were the responsibility of the institutions that commissioned, funded, or carried out the DHS surveys.

### Selection of Indicators

We examined a diverse set of essential preventative and curative coverage indicators including the following: family planning needs satisfied (FPS), antenatal care with a skilled provider (ANCS), 4 or more antenatal care visits (ANC4), skilled attendant at birth (SBA), early initiation of breastfeeding (within 1 hour) (EIBF), 3 doses of diphtheria-tetanus-pertussis vaccine (DPT3), measles vaccination (MSL), full immunization of children (FULL), vitamin A supplementation (within 6 months) (VITA), oral rehydration therapy (ORT) and continued feeding for children with diarrhea, and care seeking for children with pneumonia (CPNM). Indicators selected for detailed subanalysis were those that represented opposite ends of the continuum of care and had diverse delivery strategies (ie, health systems based, outreach focused, or community led). All indicators were defined as per the Countdown to 2015 guidelines^1^ and have been detailed in the eTable in the Supplement.

### Composite and Co-coverage

We analyzed 2 summary measures of coverage, the composite coverage index (CCI)^17^ and the co-coverage indicator.^18^ These widely used complementary indices are useful for within- and between-country comparisons, and for measuring change over time.^6^ An aggregated index, the CCI is an equally weighted average of 4 stages of interventions across the continuum of care (eTable in the Supplement): family planning, maternal and newborn care, immunization, and case management of sick children. The co-coverage index is measured at the individual or family level and includes the following: ANCS, 2 doses of tetanus toxoid during pregnancy, SBA, VITA, BCG vaccine (vaccine for tuberculosis), DPT3, MSL, access to improved drinking water, and use of an insecticide-treated bed net for children. Co-coverage is calculated as the proportion of essential interventions received by a mother and child pair, ranging from 0 (being no interventions received) to 9 (being 100% of interventions received). We also reported co-coverage with 6 or more preventive interventions (CC6+) by mother and child pair.

### Measures of Inequality

We estimated socioeconomic position using the wealth score derived from Principals Components Analysis applied to household asset data.^19^ The creation of asset indices is considered to be more reliable than using a single-proxy measure for socioeconomic position, such as maternal education or place of residence, and is a method that has been widely adopted for use in low- and middle-income countries.^20^ Where sample size permitted, coverage indicators were single- and double-disaggregated by wealth quintiles (quintiles 1-5 of the asset score) and urban and rural residence.

Equiplots that show the distance in coverage between various population strata (eg, wealth quintiles) are useful to determine patterns of inequality, including linear, top, and bottom inequality, which can then be used for appropriate targeting of interventions.^6^ Linear inequality exists when the distance between each estimate is equal, whereas top inequality represents a situation where the widest gap exists for the highest quintile and the opposite is true of bottom inequality.^6^

Equity literature stresses the importance of examining both absolute and relative inequalities which are complementary and together reveal the full picture of disparities.^6,7^ Absolute inequality highlights the actual coverage gap that exists between extreme wealth groups and the corresponding efforts that are required to close it. Relative inequality shows the degree of unfairness between the richest and the poorest. We calculated both simple and sophisticated measures for both absolute and relative inequality. Simple measures are useful for conveying messages to the lay-audience (eg, policymakers in Kenya), although they incorporate only the top (quintile 5) and bottom (quintile 1) quintiles of the population. Sophisticated measures use the full data distribution (quintiles 1-5) and thus more accurately show the magnitude of metrics. Absolute inequalities were evaluated using the basic gap between extreme quintiles (quintile 5 minus quintile 1) and the slope index of inequality (SII). Relative inequalities were estimated using the relative ratio (quintile 5 to quintile 1) and the concentration index (CIX). The SII was interpreted as the percentage point difference between the rich and poor, where greater values correspond to the intervention having higher coverage in the wealthier subgroup and 0 implying absence of inequality. The CIX is related to the Gini coefficient, which is a widely used summary measure to judge income inequality in a given country.^6^ The Gini index will equal 0 in a society that is perfectly equal in terms of income.^6^ Similarly, CIX values fall between −1 and 1, where negative values imply higher intervention coverage among the poor, positive values imply higher coverage among the rich, and 0, again, indicates the absence of inequality. For easier interpretation, the CIX values were multiplied by 100. The CIX (values) and SII (%) were also grouped into low (<15), moderate (15-40), high (40-60), and very high (>60) categories of socioeconomic inequality.^7^ The SII and CIX were calculated with standard errors and 95% CIs, using standardized methods.^6,21^

### Regional Analyses

Where sample size permitted, analyses of intervention coverage and inequalities from Kenya’s 2014 DHS were disaggregated into 8 regions, 47 counties, and 290 subcounties (constituencies). Socioeconomic inequality patterns were examined across Kenya’s 8 regions—central, coast, eastern, Nairobi, north eastern, Nyanza, Rift Valley, and western—where adequate sample size ensured statistical power of derived estimates. Prior to introduction of a devolved government under the constitution change in 2010, these regions constituted administrative provinces. The SII and CIX were examined for SBA, MSL, and CC6+ indicators.

To examine geospatial patterns in RMNCAH intervention coverage across the nation, county and constituency level estimates were calculated. The CCI was estimated for Kenya’s 47 counties. Given that the K-DHS 2014 was not powered for subcounty estimates, Bayesian small area estimation spatial models^22,23,24^ were used to generate constituency level coverage for key RMNCAH indicators. Constituency level estimates were generated for 2 key socioeconomic indicators given their importance in health care service use and care-seeking behavior of the family; household poverty (% households in the 2 poorest wealth quintiles) and maternal illiteracy.

Data analysis was conducted from April 2018 to November 2018. All analyses were carried out in Stata, version 12.0 (Stata Corp) and Arc Map 9.3 was used to create high-resolution country maps for CCI.

## Results

### Coverage and Absolute and Relative Inequality

**Figure 1.**
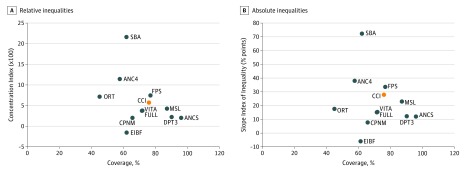
Comparisons of Relative and Absolute Inequality Between 10 Preventive Interventions, Plus the Composite Coverage Index (CCI) ANC4 indicates 4 or more antenatal care visits; ANCS, antenatal care with a skilled clinician; CPNM, care seeking children with pneumonia; DPT3, 3 doses of diphtheria-tetanus-pertussis vaccine; EIBF, early initiation of breastfeeding (within 1 hour); FPS, family planning needs satisfied; FULL, full immunization of children; MSL, measles vaccination; ORT, oral rehydration therapy; SBA, skilled attendant at birth; and VITA, vitamin A supplementation (within 6 months).

For this analysis, representative samples of 31 380 women of reproductive age and 29 743 children younger than 5 years from across Kenya were included. Table 1 shows the average national coverage levels for 11 essential interventions across the continuum of care, along with their absolute and relative inequalities in 2014. Figure 1 shows each intervention’s coverage levels contrasted with relative and absolute inequalities (ie, CIX and SII, respectively). Nationally, intervention coverage ranges from 45.1% ( 95% CI, 42.4%-47.9%) to 95.9% (95% CI, 95.4%-96.4%) across the continuum of care. Indicators with lowest coverage were ORT (45.1%; 95% CI, 42.4%-47.9%) and ANC4 (57.6%; 95% CI, 56.2%-59.0%), while ANCS (95.9%; 95% CI, 95.4%-96.4%), DPT3 (90.1%; 95% CI, 88.7%-91.5%), and MSL (87.1%; 95% CI, 85.7%-88.5%) showed the highest coverage. All interventions revealed pro-rich inequalities, except for early breastfeeding where coverage levels were slightly higher among the poor (quintile 1: 66.7%; 95% CI, 62.9%-70.2% and quintile 5: 58.9%; 95% CI, 51.8%-65.5%).

**Table 1.** Coverage and Magnitude of Inequalities by Intervention in 2014

Intervention	% (95% CI)					Value Ratio (Q5:Q1)	Concentration Index (×100), % (95% CI)
	Overall Coverage	Quintile 1 Coverage	Q5 Coverage	Difference (Q5 Minus Q1, Percentage Points)	Slope Index of Inequality, Pecentage Points		
Family planning needs satisfied	76.84 (75.31 to 78.38)	52.11 (47.93 to 56.28)	85.52 (82.86 to 88.18)	33.41 (31.90 to 34.93)	33.61 (28.51 to 38.70)	1.64	7.48 (6.31 to 8.65)
Antenatal care with a skilled provider	95.89 (95.40 to 96.36)	89.67 (88.08 to 91.25)	98.97 (98.40 to 99.54)	9.30 (8.29 to 10.32)	11.90 (9.49 to 14.32)	1.10	1.96 (1.62 to 2.29)
Antenatal care visits (≥4 visits)	57.60 (56.20 to 59.00)	43.95 (41.46 to 46.45)	74.98 (73.01 to 76.95)	31.03 (30.5 to 31.55)	38.03 (34.40 to 41.65)	1.71	11.45 (10.31 to 12.59)
Skilled birth attendant	61.85 (59.89 to 63.81)	31.12 (28.29 to 33.95)	92.70 (91.35 to 94.05)	61.58 (60.10 to 63.06)	72.47 (69.65 to 75.29)	2.98	21.68 (20.47 to 22.88)
Early start of breastfeeding	29.74 (28.70 to 30.78)	31.41 (29.36 to 33.47)	28.16 (24.89 to 31.43)	−3.25 (−4.47 to −2.04)	−1.59 (−5.41 to 2.24)	0.90	−1.29 (−3.40 to 0.83)
DPT3 immunization	90.09 (88.72 to 91.47)	83.65 (80.73 to 86.58)	92.84 (89.26 to 96.42)	9.19 (8.53 to 9.84)	12.26 (6.67 to 17.85)	1.11	2.20 (1.23 to 3.18)
Measles immunization	87.08 (85.70 to 88.46)	76.36 (73.45 to 79.26)	93.06 (90.00 to 96.12)	16.7 (16.55 to 16.86)	22.94 (17.70 to 28.18)	1.22	4.23 (3.29 to 5.18)
Full immunization	71.34 (69.22 to 73.46)	61.99 (58.30 to 65.69)	73.15 (66.21 to 80.09)	11.16 (7.91 to 14.4)	15.33 (6.91 to 23.75)	1.18	3.76 (1.81 to 5.72)
Vitamin A in past 6 mo	71.74 (70.45 to 73.04)	64.26 (61.69 to 66.84)	76.44 (73.57 to 79.30)	12.18 (11.88 to 12.46)	15.37 (10.97 to 19.78)	1.19	3.79 (2.77 to 4.82)
Oral rehydration therapy	45.13 (42.36 to 47.90)	39.21 (35.29 to 43.13)	54.52 (47.00 to 62.04)	15.31 (11.71 to 18.91)	17.69 (9.41 to 25.96)	1.39	7.16 (4.20 to 10.12)
Care seeking for pneumonia	65.74 (62.69 to 68.79)	62.56 (57.40 to 67.71)	73.49 (65.35 to 81.62)	10.93 (7.95 to 13.91)	7.78 (−2.59 to 18.14)	1.17	2.03 (−0.56 to 4.61)
Composite coverage index	75.84 (62.26 to 84.98)	62.26 (61.28 to 63.22)	84.98 (84.04 to 85.91)	22.72 (22.76 to 22.68)	5.7 (2.2 to 9.1)	1.36	26.9 (21.7 to 32.1)

Abbreviations: DPT3, diphtheria-pertussis-tetanus; Q1, lowest quintile of household wealth; Q5, highest quintile of household wealth.

Absolute gaps were greatest for ANC4, FPS, and most notably for SBA, where both the absolute difference (quintile 5 minus quintile 1: 61.6% ; 95% CI, 60.1%-63.1%) and SII (72.5%; 95% CI, 69.7%-75.3%) summary measures were highest. Using relative inequality indices, SBA (quintile 5 to quintile 1 ratio: 2.98; CIX, 21.68%; 95% CI, 20.47%-22.88%) again was the most inequitable, followed by ANC4 (quintile 5 to quintile 1 ratio: 1.71; CIX, 11.5%; 95% CI, 10.3%-12.6%). This translates to coverage that is 3.0 times and 1.7 times greater in the richest quintile when compared with the poorest for SBA and ANC4, respectively. When coverage is plotted against inequality (Figure 1), it is evident that the most inequitable interventions (absolute difference in coverage between the wealthiest [quintile 5] and poorest quintiles [quintile 1], SBA: 61.6%; 95% CI, 60.1%-63.1%, FPS: 33.4%; 95% CI, 31.9%-34.9%, and ANC4: 31.0%; 95% CI, 30.5%-31.6%) have moderate to high levels of coverage with FPS having particularly high-coverage levels (76.8%; 95% CI, 75.3%-78.4%).

The most equitable intervention across all 4 equality measures was EIBF, which exhibited minimal to no inequality (in fact, inequality that was present was pro-poor). Given EIBF’s relatively low coverage overall (nationally: 62.2%; 95% CI, 60.0%-64.4%), these findings suggest that almost 40% of children across all wealth quintiles are not being put to the breast on time. Both DPT3 and ANCS were also fairly equitable, showing an absolute difference of 9.2% (95% CI, 8.5%-9.8%) and 9.3% (95% CI, 8.3%-10.3%), respectively, and a ratio of 1.1 for both, indicating that coverage of these 2 interventions is only 1.1 times greater in quintile 5 compared with quintile 1. Both DPT3 and ANCS demonstrated the best combination of high coverage and low inequalities among all 11 interventions examined.

Similar to DPT3, overall coverage of MSL was high, although receipt of this vaccine showed greater inequality than both DPT3 and FULL. The absolute difference in coverage of MSL between the rich and poor quintiles was 16.7% (95% CI, 16.6%-16.9% and SII: 22.9%; 95% CI, 17.7%-28.2%) and coverage was 1.2 times greater in Q5 compared with Q1 (CIX: 4.2%; 95% CI, 3.3%-5.2%). The other interventions demonstrated variable levels of coverage and inequality.

### Patterns of Inequality

**Figure 2.**
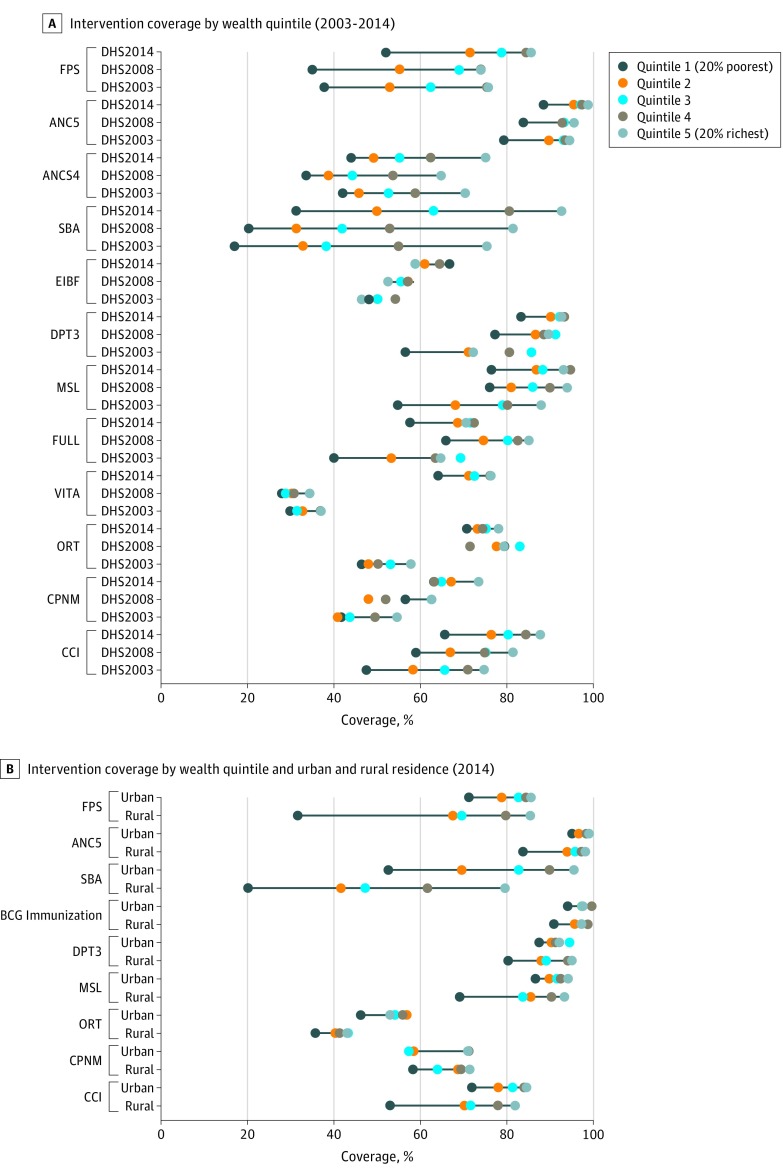
Five-Dot Chart Showing Intervention Coverage and Composite Coverage Index by Wealth Quintile From 2003 to 2014 and by Wealth Quintile and Urban and Rural Residence in 2014 Republished under the Creative Commons Attribution (CC-BY 4.0).^2^ ANC indicates antenatal care; ANC4, 4 or more antenatal care visits; ANCS, antenatal care with a skilled clinician; BCG, Bacillus Calmette–Guérin; CCI, composite coverage index; CPNM, care seeking children with pneumonia; DHS, demographic and health survey; DPT3, 3 doses of diphtheria-tetanus-pertussis vaccine; EIBF, early initiation of breastfeeding (within 1 hour); FPS, family planning needs satisfied; FULL, full immunization of children; MSL, measles vaccination; ORT, oral rehydration therapy; SBA, skilled attendant at birth; and VITA, vitamin A supplementation (within 6 months).

Figure 2A shows trends in intervention coverage by wealth quintile. For most interventions, as expected, populations in quintile 5 had greater coverage than quintile 4, and so on to quintile 1. Where dots are not connected with a line indicates a pattern in the unexpected direction (ie, coverage in a lower quintile was higher than coverage in a higher quintile).

Trends from 2003 to 2014 showed that coverage within each wealth quintile increased over time, with the exception of FULL, which showed an overall decline in coverage from 2008 to 2014. However, for most interventions, patterns of inequality remained relatively consistent. In 2014, most interventions, including FPS, ANCS, DPT3, VITA, MSL, and FULL showed bottom inequality, whereby coverage among the poorest populations was lagging behind coverage in any other quintile. Top inequality was noted for CPNM and ANC4, whereas SBA and ORT showed linear inequality. In several cases, such as for FPS, MSL, VITA, and FULL it appears that a bottom inequality pattern has become more pronounced over time.

Given the expanse of urban slums in Kenya (ie, urban poor), we double-disaggregated indicators by wealth and urban and rural residence to dig deeper into the true burden of poor coverage (Figure 2B). For almost all interventions in both urban and rural settings, a bottom inequality pattern was noted, although this pattern was decidedly pronounced for FPS and MSL among rural families. For most interventions, with the exception of SBA and ORT, coverage within quintile 2 to quintile 5 was similar in urban and rural settings indicating that the once “urban advantage” has since dissipated and targeting should focus on the poorest populations, regardless of place of residence.

### Composite and Co-coverage Estimates

The composite coverage across 8 essential interventions in Kenya was 75.8% (95% CI, 62.3%-85.0%) in 2014, indicating that a quarter of the population was not receiving adequate coverage. The CII displayed moderate levels of relative inequality and fairly high levels of absolute inequality (Figure 1). The absolute gap between Q5 and Q1 was 22.7% (95% CI, 22.8%-22.7%) and coverage was 1.36 times greater in the richest relative to the poorest quintile. The CCI showed improvements in coverage for each wealth quintile by year, although showed a bottom inequality pattern consistently (Figure 2). In 2014, the corresponding CCI coverage gaps across wealth quintiles were 34.4%, 23.7%, 19.7%, 15.6%, and 12.2% for Q1 to Q5 (eFigure 1 in the Supplement). This indicates that more efforts need to be made especially among the poor to reach universal coverage.

The study also examined co-coverage or the proportion of mother and child pairs who received 0 to 9 essential interventions by wealth quintile (eFigure 2 in the Supplement). Inequalities were noted across the wealth quintiles, with the poorest families receiving the lowest number of interventions. Among mother and child pairs in the lowest quintile, 50% received 2 interventions or less and approximately 10% received no intervention at all. In contrast, only 7% of mother and child pairs in the highest quintile received 2 interventions or less and everyone in quintile 5 received at least 1 intervention. The proportion that received all 9 interventions was low across all wealth quintiles reaching only 2.5% among the richest.

### Regional Inequalities

**Figure 3.**
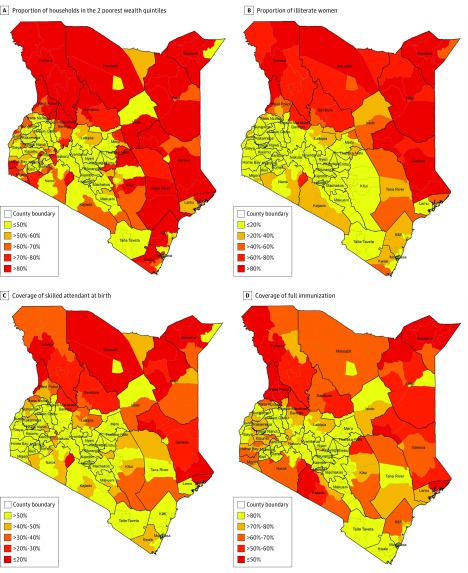
The Proportion of Households in the 2 Poorest Wealth Quintiles, the Proportion of Illiterate Women, Coverage of Skilled Birth Attendance, and Coverage of Full Immunization Mapped At the Subcounty (Constituency) Level in 2014

Figure 3 shows the constituency-level geospatial patterns of household wealth, maternal illiteracy, SBA, and FULL coverage. Examining the maps collectively revealed palpable disparities in the northern and eastern regions of Kenya, especially when compared with Nairobi. In contrast to poverty, which is widespread throughout the country but shows some subcounty differences (Figure 3A), there is a clear separation of literate and illiterate women by county, with illiteracy affecting over 60% (and >80% in many areas) of all women in Garissa, Wajir, Mandera, Marsabit, Samburu, and Turkana counties (Figure 3B). Coverage of SBA (Figure 3C) was low throughout Kenya, but lowest (≤20%) in areas of high poverty and illiteracy. Similar coverage patterns were noted for FULL (Figure 3D), with additional constituencies in the western and Rift Valley regions experiencing particularly low levels of vaccinations (≤50%). All other preventive interventions (FPS, ANC, and ANC4+) showed similar patterns of inequalities by geography (eFigures 3A-C in the Supplement). In contrast, coverage of ORT did not follow any particular regional pattern (eFigure 3D in the Supplement), while CPNM was lower in areas of low wealth and high illiteracy (eFigure 3E in the Supplement). All 4 maps highlight the regional differences that are evident even between neighboring constituencies. For example, Saku constituency in Marsabit county had visibly higher SBA coverage (>50% vs ≤20%), FULL coverage (>80% vs 60%-70%), and lower rates of illiteracy (<60% vs >80%) when compared with its surrounding regions. This constituency houses the county headquarters and represents the urban center of Marsabit town, while the other areas are composed mostly of desert and mountain ranges.

Using composite coverage as an aggregate indicator, we mapped CCI by county (eFigure 4 in the Supplement) and found major geospatial differences that aligned with our constituency maps. Along with West Pokot (Rift Valley region), counties that composed the north eastern region are substantially worse off. The CCI coverage is 60% or less within Kenya’s coast and eastern regions as well. In contrast, Nairobi and central regions showed the highest coverage of CCI (>70%).

We analyzed coverage and inequalities in SBA, MSL, and CC6+ by larger geographical regions in Kenya. Coverage estimates are presented in Table 2 while eFigures 5A-C in the Supplement visualize the wealth inequality indices (SII vs CIX) for each indicator.

**Table 2.** Coverage of Skilled Birth Attendance, Measles Immunization, and Co-coverage (≥6 Preventive Interventions) in 8 Regions of Kenya

Region	% (95% CI)		
	Skilled Birth Attendance	Measles	Co-coverage (≥6 Preventive Interventions)^a^^,^^b^
Coast	58.24 (52.68-63.80)	86.61 (82.56-90.67)	23.09 (20.37-25.82)
North eastern	32.36 (23.57-41.14)	69.85 (61.65-78.04)	10.06 (7.69-12.43)
Eastern	63.30 (58.76-67.83)	92.09 (88.79-95.39)	22.21 (19.71-24.72)
Central	89.73 (87.62-91.83)	97.16 (95.34-98.98)	27.27 (23.53-31.01)
Rift Valley	51.27 (47.83-54.70)	83.14 (80.57-85.72)	16.51 (15.11-17.91)
Western	47.79 (43.83-51.75)	85.74 (80.93-90.55)	20.97 (18.87-23.07)
Nyanza	65.04 (61.20-68.88)	85.26 (81.66-88.86)	21.77 (19.42-24.12)
Nairobi	89.10 (84.71-93.49)	92.52 (87.85-97.19)	26.13 (22.23-30.04)

^a^ Preventive interventions: antenatal care with a skilled provider, 2 doses of tetanus toxoid during pregnancy, skilled birth assistance, child supplementation with vitamin A, BCG vaccine, diphtheria-pertussis-tetanus vaccine, measles vaccine, access to improved drinking water, or use of an insecticide-treated bed net for children.

^b^ Co-coverage, 6 or more, represents the proportion of mother and child pairs who received 6 or more preventive interventions.

Across all regions examined, CC6+ was low ranging from 10% in the north eastern region to 27% in the Nairobi region (Table 2). Conversely, MSL coverage was high, although coverage in the north eastern region lagged behind the rest. Skilled birth attendance showed variable coverage, with very poor use in the north eastern region (32.3%; 95% CI, 23.6%-41.1%) and high use in central (89.7%; 95% CI, 87.6%-91.8%) and Nairobi (89.1%; 95% CI, 84.7%-93.5%) regions. For all other regions, coverage of this intervention was 65% or less.

Of the 3 indicators examined, SBA demonstrated the greatest variation in both absolute and relative inequality by region (eFigure 5A in the Supplement), underscoring major geospatial differences for this intervention. Kenya’s eastern, coast, Rift Valley, and north eastern regions all showed high absolute inequality, although the north eastern region was the only area that also displayed high relative inequality. Similarly, for MSL, both absolute and relative inequalities were highest in the north eastern region while Nairobi and Central regions showed the least inequality for both SBA and MSL (eFigure 5A and B in the Supplement). When examining CC6+, we did not note many regional differences (eFigure 5C in the Supplement); a finding that likely reflects low mean coverage overall. However, the north eastern region demonstrated the highest relative inequality, whereas Nairobi and Rift Valley regions had the highest absolute inequality. Results clearly show the north eastern region to be the outlier for each indicator examined.

## Discussion

To our knowledge, this is the first effort to comprehensively examine inequalities in a range of maternal and child health indicators at a subnational level in Kenya. The results of this study have revealed that socioeconomic and regional inequalities exist, underscoring the reality that certain population groups are being left behind in terms of health service accessibility and use.

National coverage of RMNCAH interventions in Kenya is varied. It is above 85% for ANCS, DPT3, and MSL, and is suboptimal for most other interventions. In general, we found that coverage levels were related to the ease with which the intervention could be implemented at a population level. Interventions that did not require repeated visits or access to a facility (eg, ANCS or vaccines that could be administered through mass population campaigns) had better coverage than those that required a functioning health system and multiple visits to a health care professional. Similar findings have been reported in other studies assessing inequalities in health service use across low- and middle-income countries.^7,25^ Additionally, we found that good coverage and equitable access are usually not aligned. For example, EIBF had relatively low coverage, but was found to be one of the most equitable interventions. Consequently, mass campaigns targeting women of all wealth strata should be implemented to improve uptake of this intervention. In contrast, FPS had moderately high coverage, but was found to be one of the most inequitable; a finding that suggests that mostly upper-class or upper-middle-class women are using family planning services, hence, lowest quintiles should be targeted for intervention.

Across all 4 measures examined, SBA showed the greatest inequality, followed by FPS and ANC4. Conversely, EIBF, DPT3 immunization, and ANCS were found to be most equitable. This is in line with other studies from low- and middle-income countries that have also found SBA and ANC4 to be largely inequitable and coverage of breastfeeding interventions to be similar across wealth quintiles.^7,17,25,26,27^ Interestingly, the study’s findings on ANCS appeared to be unique to the Kenyan context. Typically, ANCS tracks with SBA, meaning that high coverage and low inequality in antenatal care tends to result in a similar pattern for SBA. While this appears to be true for ANC4, we have shown that, not only is coverage of ANCS remarkably high, but it is one of the most equitable of all the interventions examined. Other studies in Kenya have found that attendance of 1 ANC visit typically does not lead to additional visits, with cited reasons being poor quality of care, leading to a limited understanding of the benefits of ANC, restrictive costs (both indirect and direct), and timing of ANC initiation.^28,29^ Pell et al^28^ noted that in Kenya only 12% of women initiated ANC in the first trimester while 40% attended their first visit in their third trimester. Considering that ANCS is already equitably distributed, herein lays a major missed opportunity for Kenya. Encouraging timely initiation of ANC, strengthening integrated approaches to maternal health service delivery, and improving the quality of care received could have important implications for furthering the uptake of associated interventions around labor, delivery, and newborn care for women of all socioeconomic strata. For example, a multicountry study, that included Kenya, has demonstrated the positive link between ANC exposure and improved facility deliveries.^30^ These findings also call for broader health systems strengthening and innovations in increasing demand-side factors to seeking care. For example, community education and mobilization initiatives (such as women’s groups), along with the engagement of household males and community elders in RMNCAH education campaigns could be considered.

Along with being pro-rich, the majority of interventions examined demonstrated a bottom-inequality pattern, indicating that the poorest 20% of the population are being left behind. This is typically reflective of relatively high-coverage countries, where only the extremely marginalized and vulnerable populations are not being reached.^18^ When double disaggregating data by wealth and urban and rural setting, we found that the bottom inequality pattern held, with the rural poor faring worse than the urban poor for most indicators. Several recent studies have highlighted faster declines in child mortality in rural areas when compared with urban settings;^31,32^ a finding that has been attributed to the proliferation of urban slums in Kenya and has garnered attention around improving health services in informal settlements. As such, our finding is important in that it underscores the need to recognize the gaps that remain for rural populations. Strategies to specifically target poor, rural families along with those residing in urban slums will be necessary to close the wide coverage gap that currently exists in Kenya. In fact, we have previously shown that the impact of community-driven interventions on newborn and under-five mortality is greatest in the poorest quintiles,^2^ confirming that a targeted approach would save the most lives.

Our geospatial work underscored palpable differences across Kenya’s various regions. There were unacceptable variations in illiteracy rates, with more than 80% of women in the northern Rift Valley and north eastern regions being illiterate compared with less than 20% in other areas. When assessing coverage of essential RMNCAH interventions, our results again pointed to populations in the north eastern region as being a clear target for intervention. Nearly three quarters of this largely insecure population live below the poverty line^33^ and more than 80% of its 3 constituent counties (Garissa, Mandera, and Wajir) are rural.^34^ Inhabitants are largely nomadic or seminomadic pastoralists, and the region was home to the Dadaab refugee camp, which contained 235 269 registered refugees and asylum seekers at the end of January 2018.^35^ Historically, the north eastern province of Kenya has felt marginalized, with repeated drought and conflict that has spilled over from Somalia. Today, the area continues to face insecurity with sporadic acts of violence incited by ethnic differences and targeted attacks by extremist group Al-Shabaab.^36^ As a result, health and social service delivery and humanitarian aid that is prevalent in other areas of Kenya has been largely depressed in these counties.^36^ Previous work has linked both inadequate health workforce and poor health facility density, which exist in this and other remote and rural regions of Kenya, with low CCI.^37^ In 2013, Mandera county had a health facility and health workforce density of 1.6 and 11.2 per 10 000 population, respectively, compared with Mombasa (a coastal city in Mombasa county) which had estimates of 3.65 and 24 per 10 000 population.^38^ This further highlights the multifaceted constitution of inequality, and the potential confounding of regional inequality by access to a functioning health system. To ensure that these vulnerable populations are reached will require a proper understanding of regional barriers, including geographical remoteness, lack of infrastructure, itinerant populations and continued insecurity. In addition, education approaches are needed to reduce rates of illiteracy; this is a strategy that will have compounding effects in terms of improving RMNCAH intervention coverage.

### Limitations

This study had limitations. First, treatment indicators (eg, ORT and CPNM) rely on maternal reporting and recall, and thus are more likely to be biased than objective indicators such as SBA or receipt of vaccination. Surveys have demonstrated differences in accurate reporting by wealth stratum,^17^ a finding that could inappropriately reduce estimated differences in coverage by socioeconomic status. Second, the use of asset indices have been criticized for the following reasons: the differential classification of households based on the assets that have been used to define the index, the strong correlation between wealth and residential inequities (because wealthy households typically reside in urban areas), and the inability to interpret absolute differences across countries, as the poorest families in a low-income country are not equivalent to those in a middle-income country.^17^ Despite these limitations, it is widely agreed that asset indices are a suitable measure of describing within-country inequalities.^6^ Additionally, we performed double disaggregation of wealth and urban and rural data in Kenya to disentangle the unique effects of each. While we have looked at wealth and residence together, we were not able to address the question of whether regional disparities noted were driven by wealth data, or vice versa. Nevertheless, we have demonstrated that both wealth and regional disparities exist in Kenya; where there are both may indicate a critical population to target.

## Conclusions

It is apparent that Kenya has made great strides in improving health service delivery for the country as a whole. Trend analysis has revealed important improvements in overall coverage of essential RMNCAH interventions from 2003 to 2014. However, we have also shown that inequalities remain persistently entrenched within the population. For Kenya to achieve universal health coverage, the most vulnerable in the country need to be reached with urgency. Targeting of urban slums, the rural poor, and especially remote communities in Garissa, Mandera, and Wajir counties will be essential. Ensuring the availability of a functioning health system and garnering a widespread focus on improving uptake of facility-based interventions would be ideal. However, where access is poor, owing to regional or other barriers, encouraging initiatives that deliver services at the community level will also help to achieve health gains in a cost-effective manner. Antenatal care with a skilled provider, which demonstrated high and equitable coverage, could be used as a unique platform in this context to encourage the uptake of other critical interventions throughout pregnancy and childbirth. In addition to a strong commitment by the current government, which has listed attainment of universal health care among its top 4 development pillars and affirmed it through several national policy and strategy documents, this will require buy-in and support from donors, nongovernmental organizations, and civil society who must work hand-in-hand to improve equality in RMNCAH services. At a time when health equity is a global focus, Kenya has the opportunity to be an example in accelerating progress in this area.

## Notes

Supplement. **eTable.** Countdown Indicators and Corresponding Definitions **eFigure 1.** Composite Coverage of Selected Interventions and Corresponding Coverage Gap (how much is needed to reach universal coverage), By Wealth Quintile **eFigure 2.** Co-coverage of Health Interventions: Percentage of Mothers and Children <5 Receiving 0 to 9 Essential Interventions By Wealth Quintile **eFigure 3.** The Coverage of Family Planning Needs Satisfied (A), Antenatal Care (ANC) With a Skilled Attendant (B), ANC 4 or More Visits (C), Early Initiation of Breastfeeding (D), Oral Rehydration Therapy for Children With Diarrhea (E), and Care Seeking for Children With Pneumonia (F), Mapped At the Subcounty (constituency) Level in 2014 **eFigure 4.** Composite Coverage (CCI) By Region, 2014 **eFigure 5.** Comparisons of Relative (concentration index, CIX) and Absolute (slope index of inequality, SII) Inequality Between Kenya’s 8 Regions for SBA (A), Measles Immunization (B), and Co-Coverage of 6 or More Preventive Interventions By Mother/Child Pair (C)
