# Supplementary material for: Assessment of Inequalities in Coverage of Essential Reproductive, Maternal, Newborn, Child, and Adolescent Health Interventions in Kenya
Source: JAMA Netw Open. 2018 Dec 28;1(8):e185152. doi: 10.1001/jamanetworkopen.2018.5152 (PMC6324360; doi:10.1001/jamanetworkopen.2018.5152)
Supplement: Supplement. — eTable. Countdown Indicators and Corresponding Definitions eFigure 1. Composite Coverage of Selected Interventions and Corresponding Coverage Gap (how much is needed to reach universal coverage), By Wealth Quintile eFigure 2. Co-coverage of Health Interventions: Percentage of Mothers and Children <5 Receiving 0 to 9 Essential Interventions By Wealth Quintile eFigure 3. The Coverage of Family Planning Needs Satisfied (A), Antenatal Care (ANC) With a Skilled Attendant (B), ANC 4 or More Visits (C), Early Initiation of Breastfeeding (D), Oral Rehydration Therapy for Children With Diarrhea (E), and Care Seeking for Children With Pneumonia (F), Mapped At the Subcounty (constituency) Level in 2014 eFigure 4. Composite Coverage (CCI) By Region, 2014 eFigure 5. Comparisons of Relative (concentration index, CIX) and Absolute (slope index of inequality, SII) Inequality Between Kenya’s 8 Regions for SBA (A), Measles Immunization (B), and Co-Coverage of 6 or More Preventive Interventions By Mother/Child Pair (C) [file jamanetwopen-1-e185152-s001.pdf]

Supplementary Online Content

Keats EC, Akseer N, Bhatti Z, et al. Assessment of inequalities in coverage of essential reproductive, maternal, newborn, child, and adolescent health interventions in Kenya. *JAMA Netw Open*. 2018;1(8):e185152. doi:10.1001/jamanetworkopen.2018.5152

eTable. Countdown Indicators and Corresponding Definitions
eFigure 1. Composite Coverage of Selected Interventions and Corresponding Coverage Gap (how much is needed to reach universal coverage), By Wealth Quintile
eFigure 2. Co-coverage of Health Interventions: Percentage of Mothers and Children <5 Receiving 0 to 9 Essential Interventions By Wealth Quintile
eFigure 3. The Coverage of Family Planning Needs Satisfied (A), Antenatal Care (ANC) With a Skilled Attendant (B), ANC 4 or More Visits (C), Early Initiation of Breastfeeding (D), Oral Rehydration Therapy for Children With Diarrhea (E), and Care Seeking for Children With Pneumonia (F), Mapped At the Subcounty (constituency) Level in 2014
eFigure 4. Composite Coverage (CCI) By Region, 2014
eFigure 5. Comparisons of Relative (concentration index, CIX) and Absolute (slope index of inequality, SII) Inequality Between Kenya's 8 Regions for SBA (A), Measles Immunization (B), and Co-Coverage of 6 or More Preventive Interventions By Mother/Child Pair (C)

This supplementary material has been provided by the authors to give readers additional information about their work.

© 2018 Keats EC et al. *JAMA Network Open*.

eTable. Countdown Indicators and Corresponding Definitions

Indicator		Definition
**ANC4**	4+ antenatal care visits	Proportion of mothers who had at least 4 antenatal care visits during last pregnancy.
**ANCS**	Antenatal care visit by skilled provider	Proportion of mothers who were seen by a skilled health provider for at least one antenatal care visit during last pregnancy.
**CCI**	Composite coverage index	CCI is calculated as the weighted average of coverage of a set of eight preventive and curative interventions; it gives equal weight to four stages in the continuum of care: family planning, maternal and newborn care, immunization, and case management of sick children. The following expression is used to obtain the estimate: where FPS is demand for family planning satisfied, SBA is skilled birth attendant, ANCS is antenatal care with skilled provider, DPT3 is three doses of DPT vaccine, MSL is measles vaccination, BCG is BCG vaccination, ORT is oral rehydration therapy and continued feeding for children with diarrhoea, and CPNM is care seeking for children with suspected pneumonia. $CCI = {}^{1}/_{4}\left(FPS + \frac{SBA+ANCS}{2} + \frac{2DPT3+MSL+BCG}{4} + \frac{ORT+CPNM}{2}\right)$
**CPNM**	Care seeking for pneumonia	Proportion of children aged 0–59 months with suspected pneumonia taken to an appropriate health provider.
**DPT3**	DPT immunization	Proportion of children aged 12-23 months who received three doses of DPT vaccine.
**EIBF**	Early initiation of breastfeeding	Proportion of newborns put to the breast in their first hour of life.
**FPS**	Family planning needs satisfied	Percentage of currently married fecund women who say that they do not want any more children or that they want to wait 2 or more years before having another child, and are using contraception (met need for contraception divided by the demand).
**FULL**	Fully immunized children	Proportion of children aged 12-23 months who received three doses of DPT and polio vaccines and one dose of BCG and measles vaccines.
**ITNC**	Insecticide treated bed net for children	Proportion of children aged 0–59 months who slept under an ITN the night before the interview.
**MSL**	Measles immunization	Proportion of children aged 12-23 months who received a dose of measles vaccine.

© 2018 Keats EC et al. *JAMA Network Open*.

**ORT**	Oral rehydration therapy	Percentage of children aged 0–59 months with diarrhea in the previous two weeks who received oral rehydration therapy (packets of oral rehydration salts, recommended home solution, or increased fluids) and continued feeding.
**SBA**	Skilled birth attendant	Proportion of mothers who had their delivery assisted by a skilled health professional.
**VITA**	Vitamin A supplementation	Proportion of children aged 6–59 months who received at least one high dose of vitamin A supplement in the previous six months.

© 2018 Keats EC et al. *JAMA Network Open*.

eFigure 1. Composite Coverage of Selected Interventions and Corresponding Coverage Gap (how much is needed to reach universal coverage), By Wealth Quintile

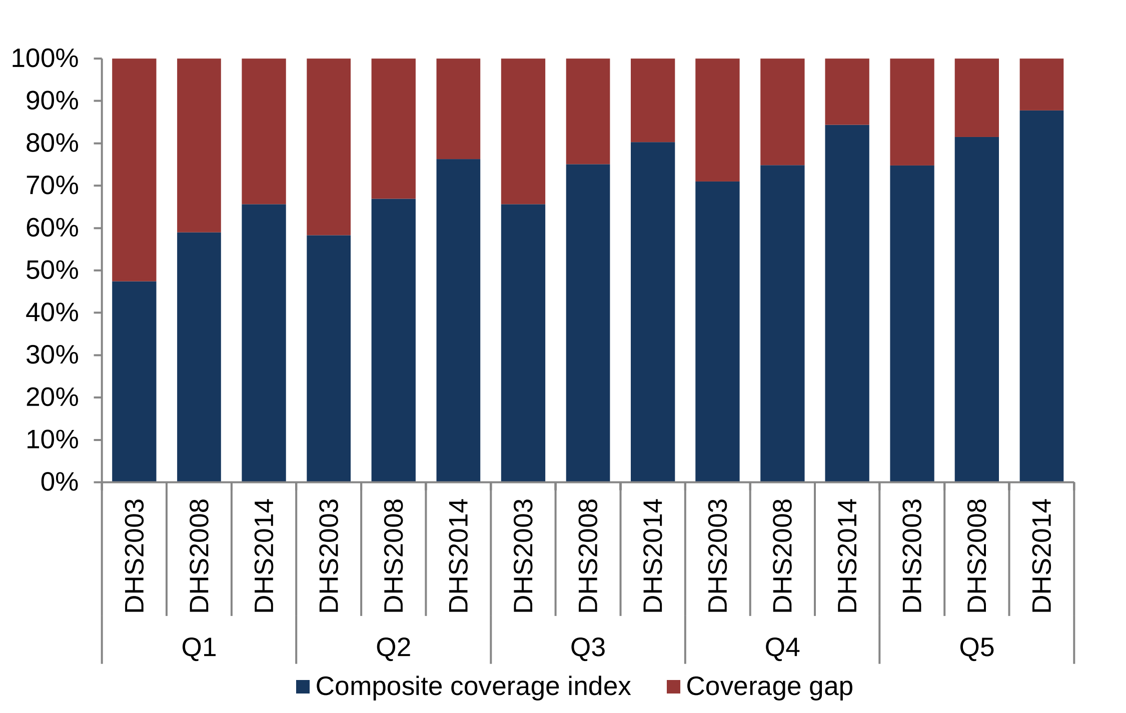

Q1=poorest 20% of population; Q5=wealthiest 20% of population.

© 2018 Keats EC et al. *JAMA Network Open*.

eFigure 2. Co-coverage of Health Interventions: Percentage of Mothers and Children <5 Receiving 0 to 9 Essential Interventions By Wealth Quintile[a]

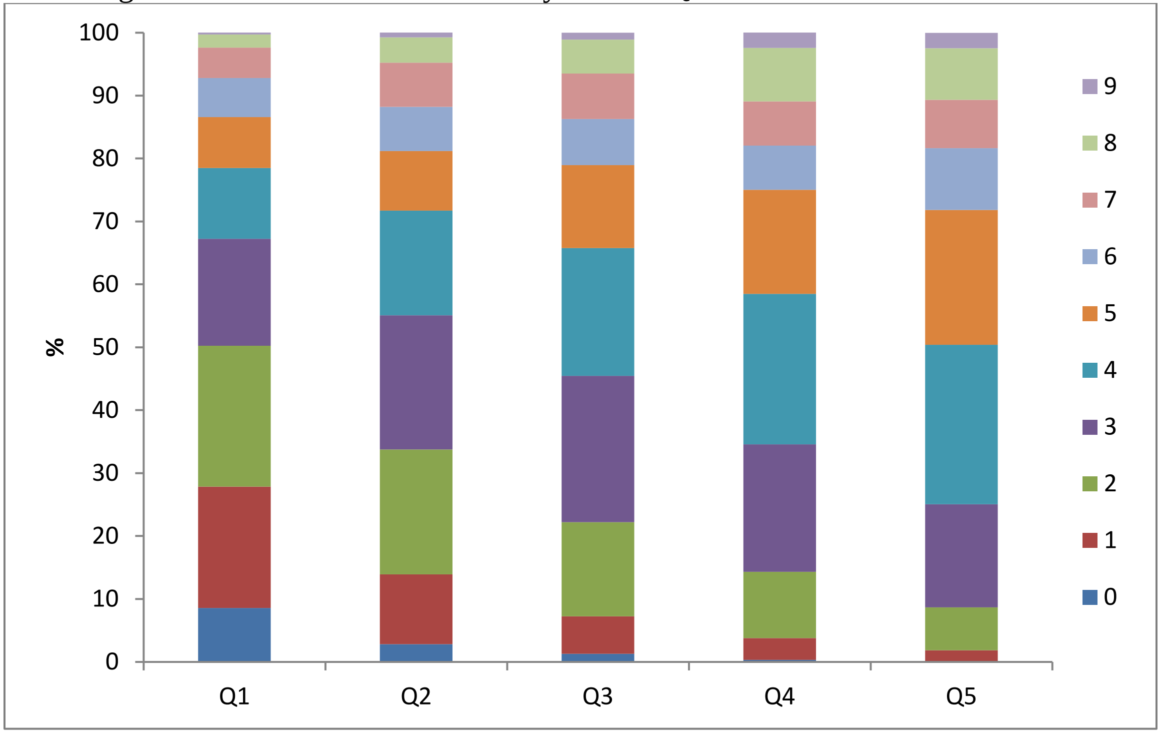

[a]Interventions taken into account for co-coverage analysis: (1) antenatal care, (2) mother immunised against tetanus, (3) skilled birth assistance, (4) BCG immunization, (5) 2 doses of DTP3, (6) measles immunization, (7) vitamin A supplementation, (8) household with improved drinking water source, (9) child slept under insecticide-treated bed net.

© 2018 Keats EC et al. *JAMA Network Open*.

eFigure 3. The Coverage of Family Planning Needs Satisfied (A), Antenatal Care (ANC) With a Skilled Attendant (B), ANC 4 or More Visits (C), Oral Rehydration Therapy for Children With Diarrhea (D), and Care Seeking for Children With Pneumonia (E), Mapped At the Subcounty (constituency) Level in 2014

eFigure 3A. Family Planning Needs Satisfied

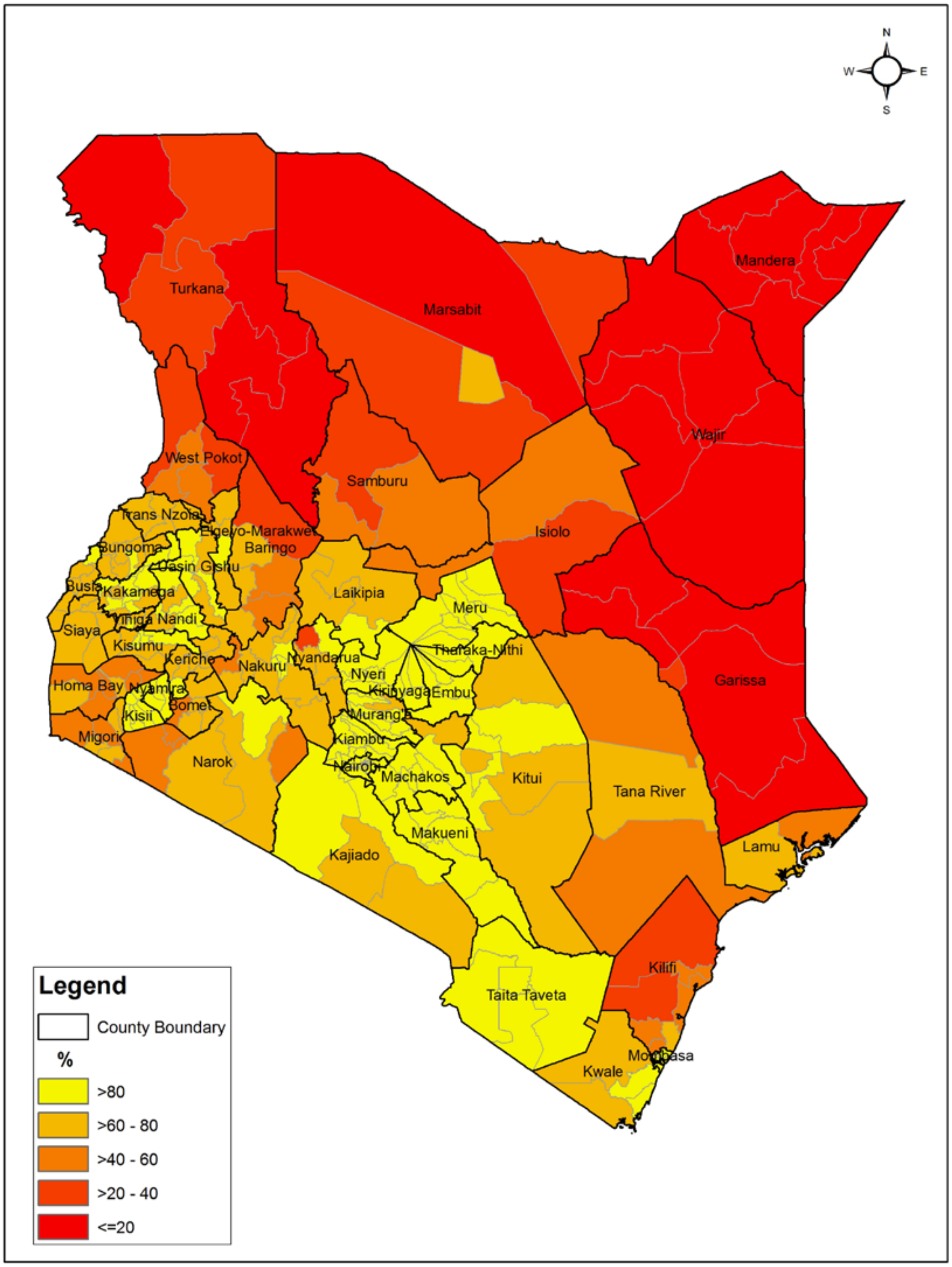

© 2018 Keats EC et al. *JAMA Network Open*.

eFigure 3B. ANC

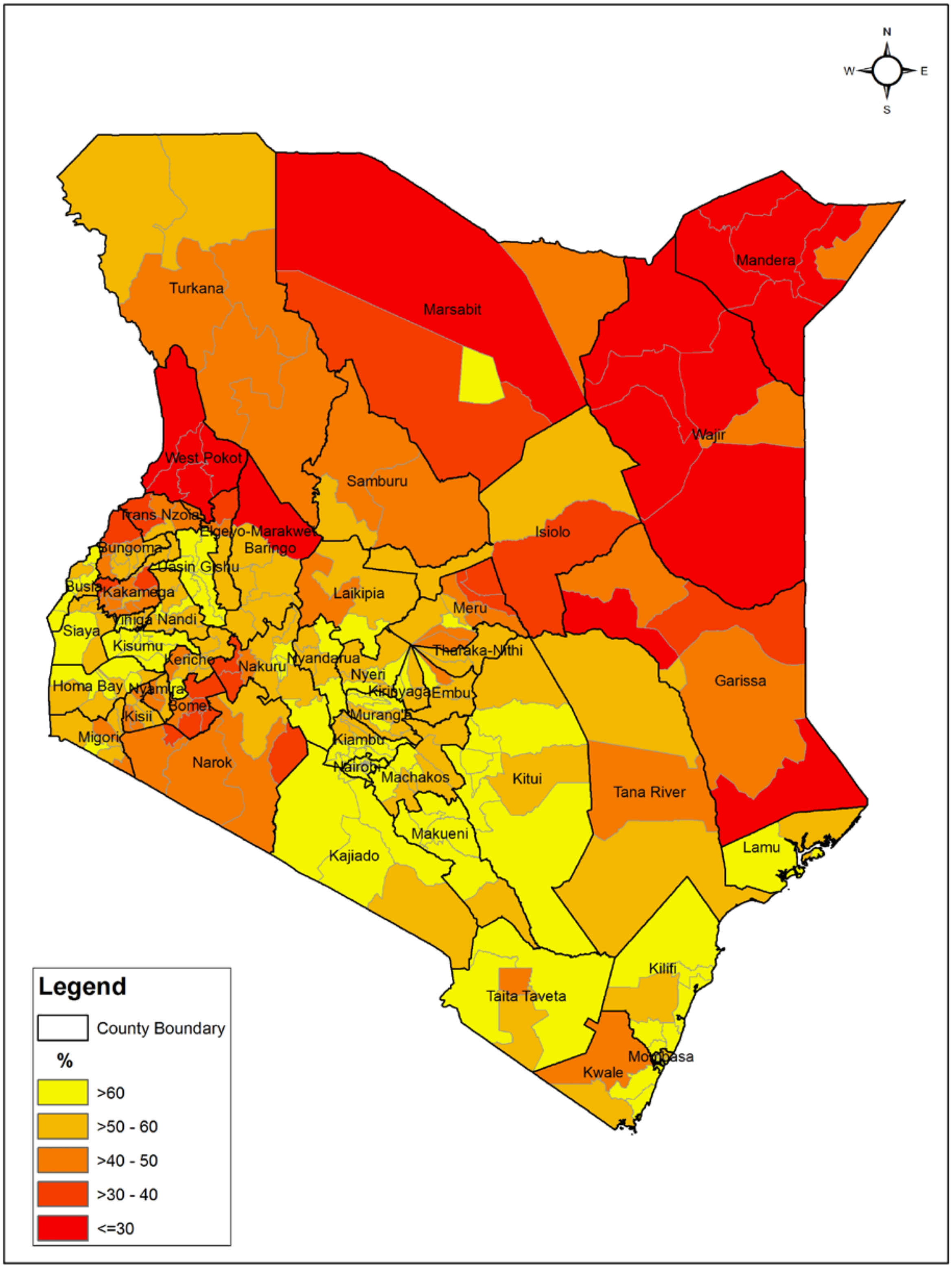

© 2018 Keats EC et al. *JAMA Network Open*.

eFigure 3C. ANC 4+

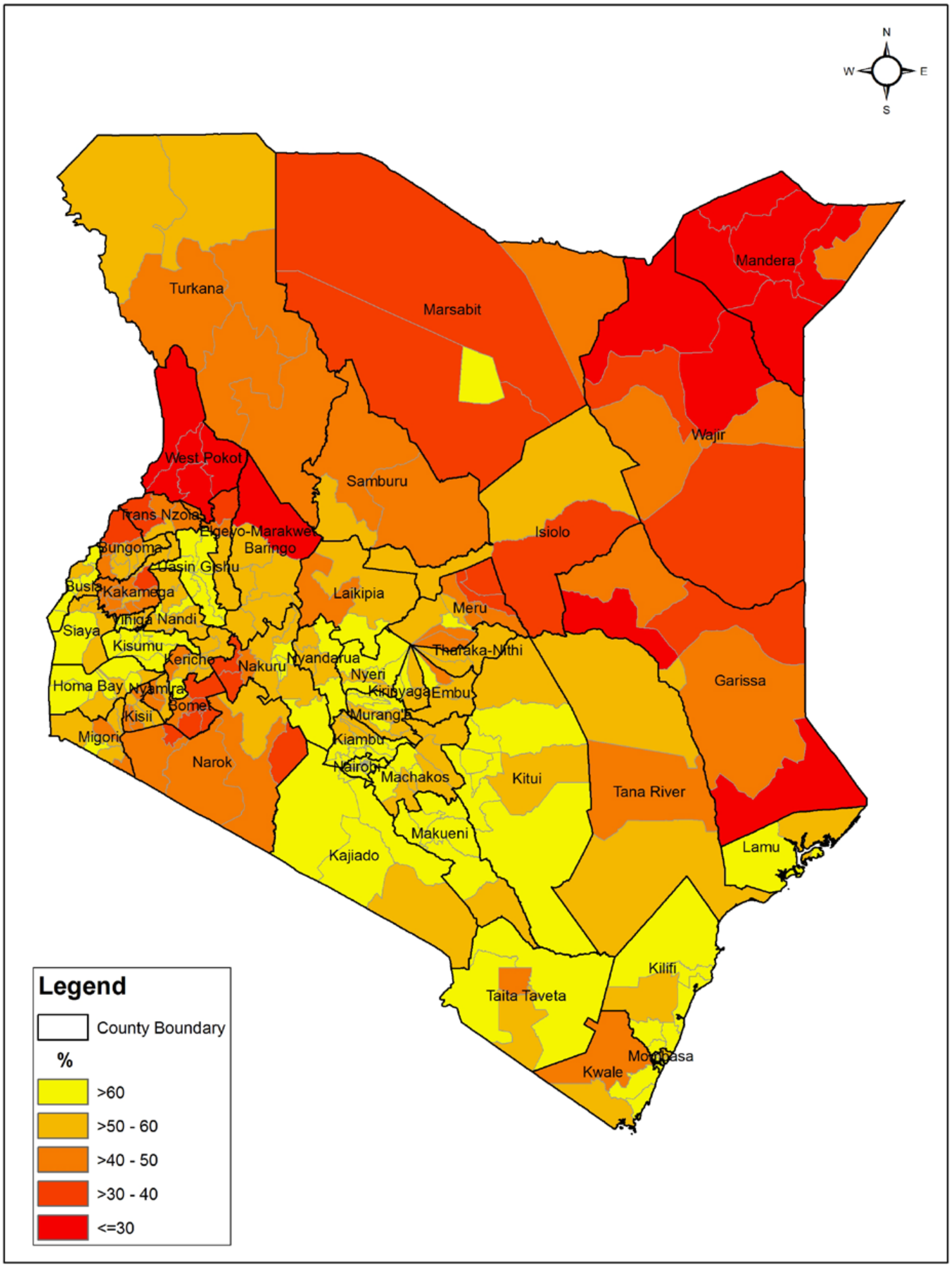

© 2018 Keats EC et al. *JAMA Network Open*.

eFigure 3D. Oral Rehydration Therapy for Children With Diarrhea

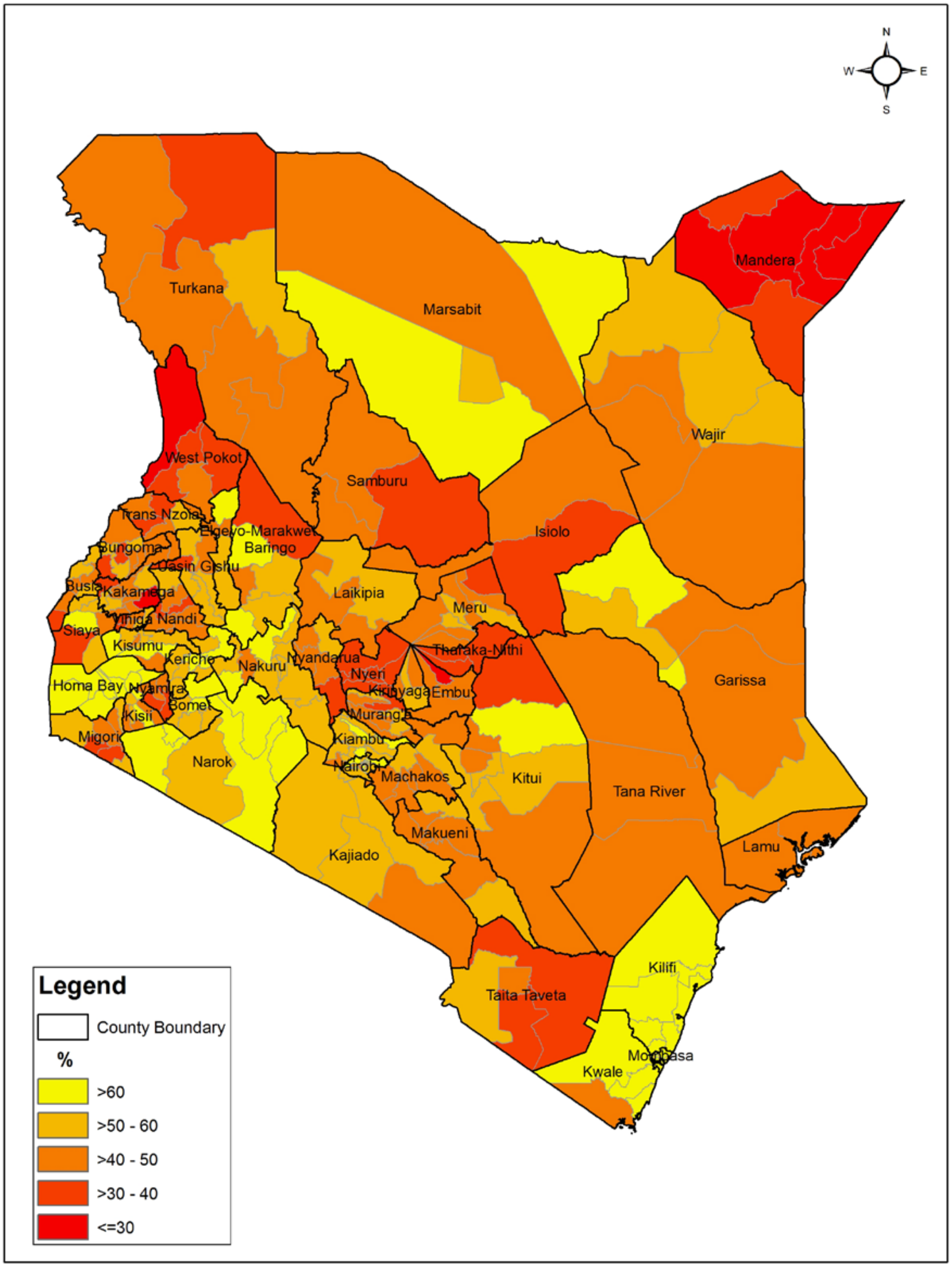

© 2018 Keats EC et al. *JAMA Network Open*.

eFigure 3E. Care Seeking for Children With Pneumonia

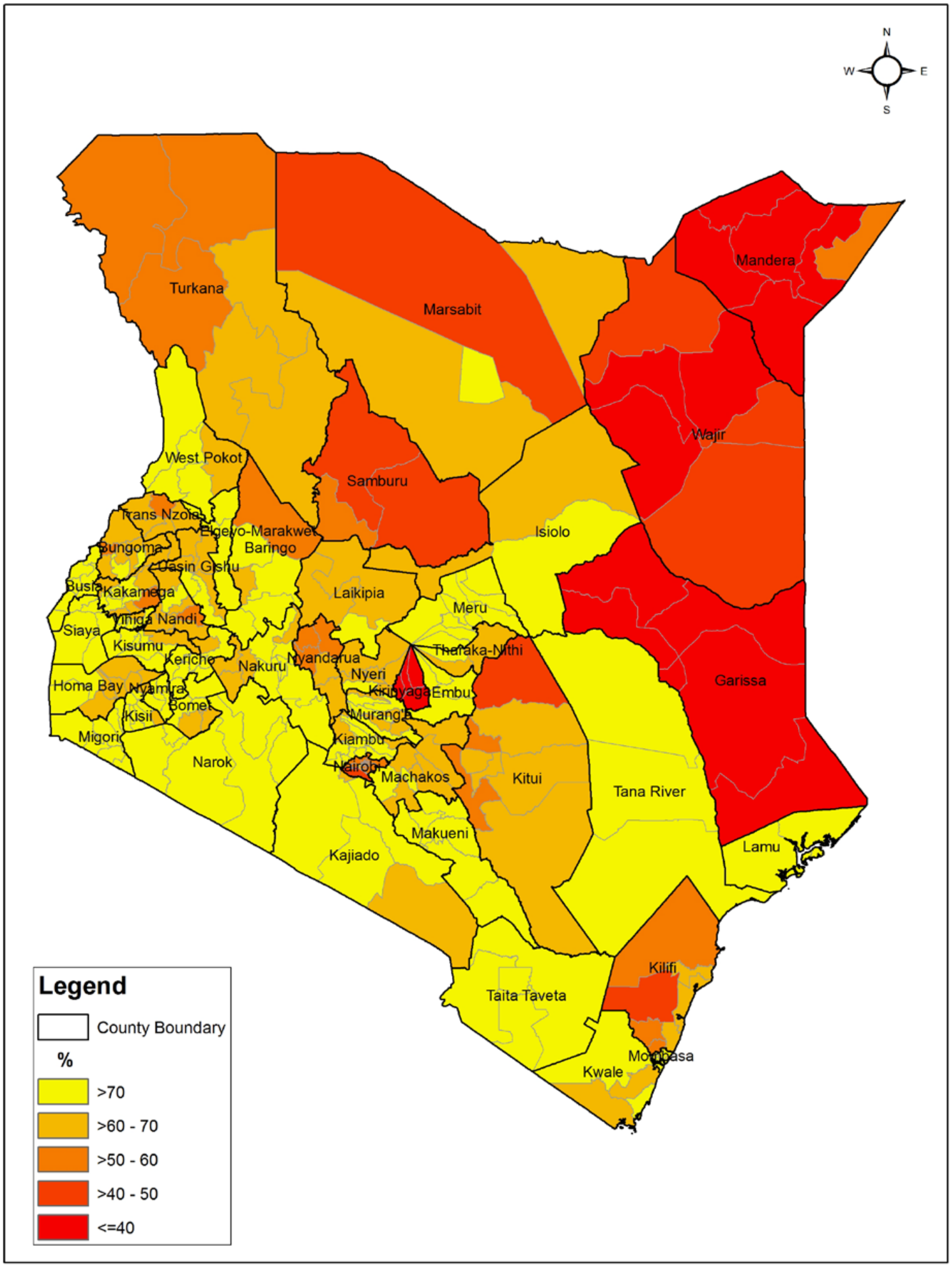

© 2018 Keats EC et al. *JAMA Network Open*.

eFigure 4. Composite Coverage (CCI) By Region, 2014

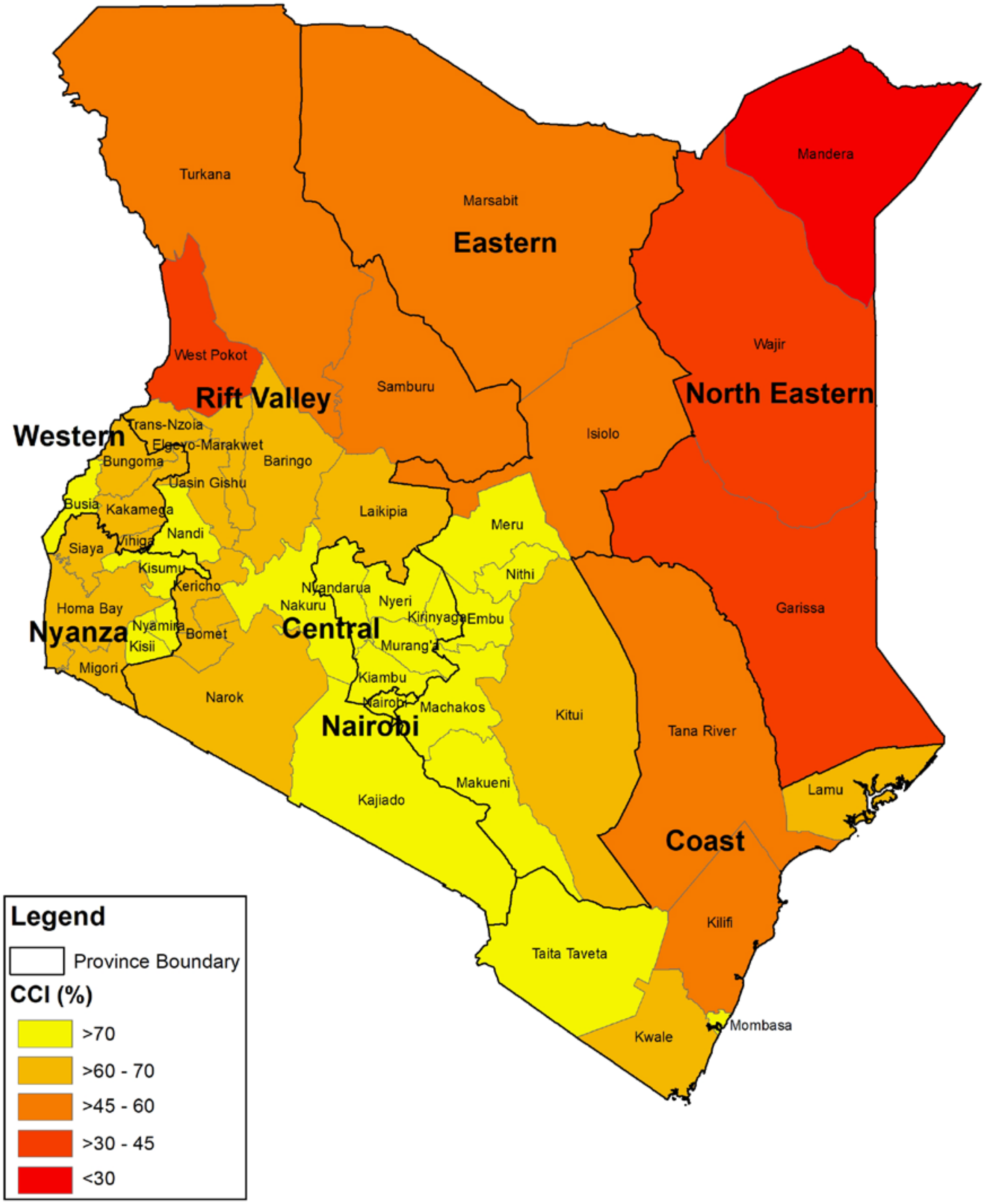

© 2018 Keats EC et al. *JAMA Network Open*.

eFigure 5. Comparisons of Relative (concentration index, CIX) and Absolute (slope index of inequality, SII) Inequality Between Kenya's 8 Regions for SBA (A), Measles Immunization (B), and Co-Coverage of 6 or More Preventive Interventions By Mother/Child Pair (C)

eFigure 5A. SBA

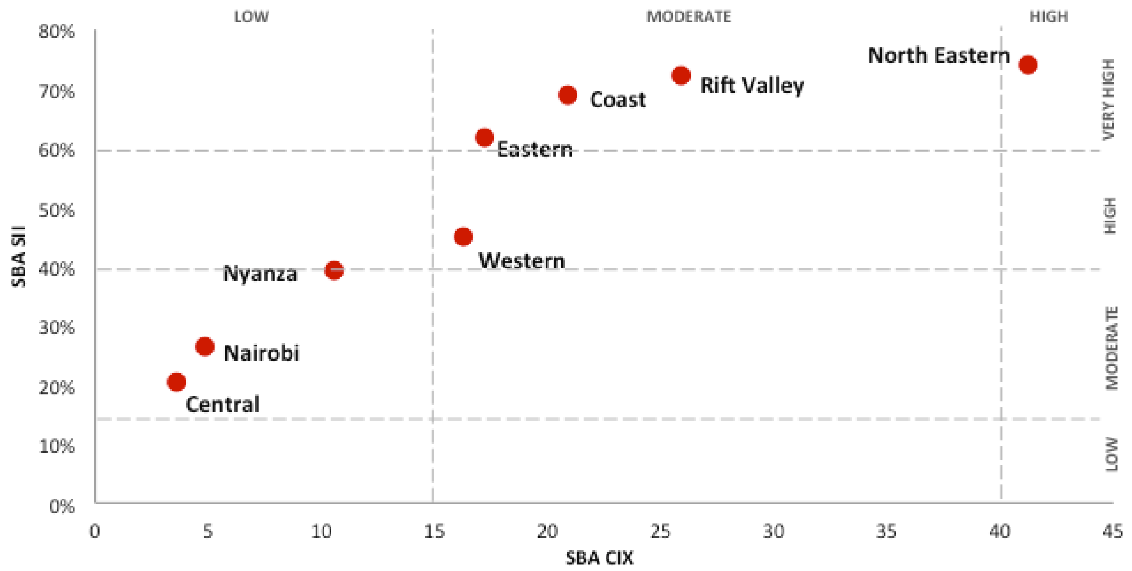

© 2018 Keats EC et al. *JAMA Network Open*.

eFigure 5B. Measles Immunization

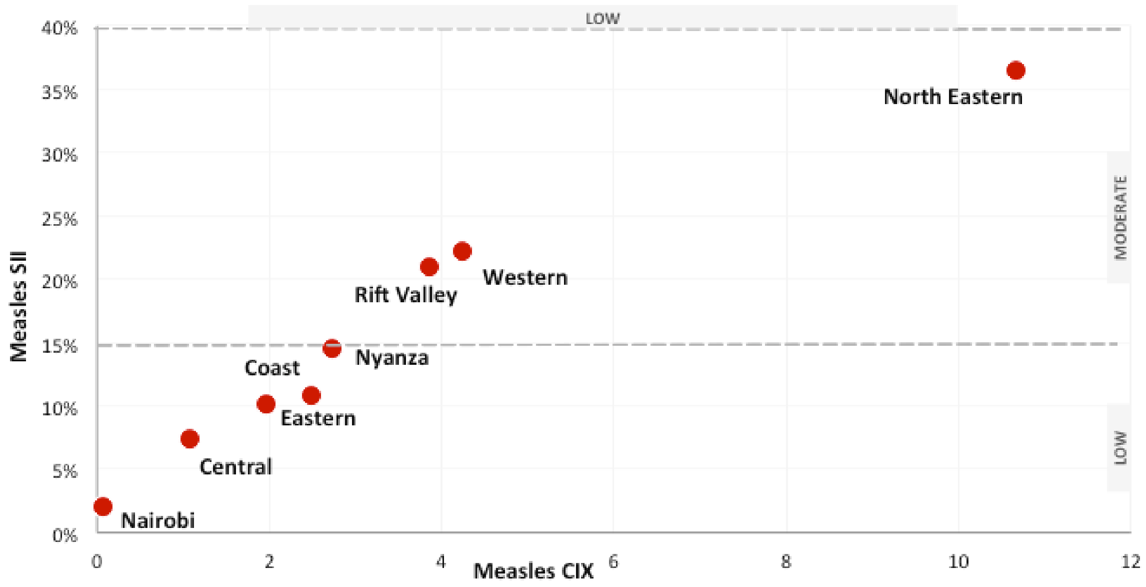

© 2018 Keats EC et al. *JAMA Network Open*.

eFigure 5C. Co-coverage of 6 or More Preventive Interventions (CC6+)

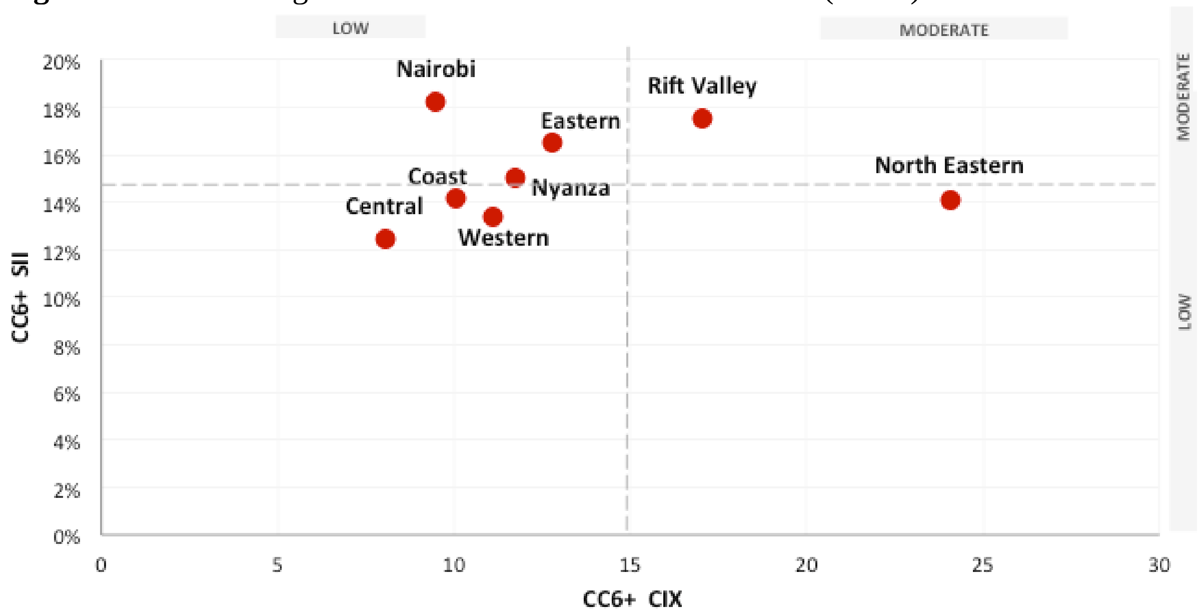

© 2018 Keats EC et al. *JAMA Network Open*.
